# Real world hospital costs following stress echocardiography in the UK: a costing study from the EVAREST/BSE-NSTEP multi-centre study

**DOI:** 10.1186/s44156-023-00020-1

**Published:** 2023-05-31

**Authors:** Casey L. Johnson, William Woodward, Annabelle McCourt, Cameron Dockerill, Samuel Krasner, Mark Monaghan, Roxy Senior, Daniel X. Augustine, Maria Paton, Jamie O’Driscoll, David Oxborough, Keith Pearce, Shaun Robinson, James Willis, Rajan Sharma, Apostolos Tsiachristas, Paul Leeson, the EVAREST/BSE-NSTEP Investigators, Jacob Easaw, Daniel X. Augustine, Abraheem Abraheem, Sanjay Banypersad, Christopher Boos, Sudantha Bulugahapitiya, Jeremy Butts, Duncan Coles, Thuraia Nageh, Haytham Hamdan, Ayyaz Sultan, Shahnaz Jamil-Copley, Gajen Kanaganayagam, Tom Mwambingu, Antonis Pantazis, Alexandros Papachristidis, Ronak Rajani, Muhammad Amer Rasheed, Naveed A Razvi, Sushma Rekhraj, David P Ripley, Kathleen Rose, Michaela Scheuermann-Freestone, Rebecca Schofield, Spyridon Zidros, Kenneth Wong, Sarah Fairbarin, Badrinathan Chandrasekaran, Patrick Gibson, Attila Kardos, Henry Boardman, Joanna d’Arcy, Katrin Balkhausen, Ioannis Moukas, Joban S Sehmi, Soroosh Firoozan

**Affiliations:** 1 Cardiovascular Clinical Research Facility, RDM Division of Cardiovascular Medicine, University of Oxford, Oxford, OX3 9DU UK; 2 Kings College Hospital NHS Foundation Hospital, London, UK; 3 Northwick Park Hospital-Royal Brompton Hospital, London, UK; 4 Royal United Hospitals Bath NHS Foundation Hospital, Bath, UK; 5 University of Leeds, Leeds, UK; 6 Canterbury Christ Church University, Canterbury, UK; 7 Research Institute for Sports and Exercise Science, Liverpool John Moores University/Liverpool Centre for Cardiovascular Science, Liverpool, UK; 8 Manchester University NHS Foundation Hospital, Manchester, UK; 9 North West Anglia NHS Foundation Hospital, Peterborough, UK; 10 St. George’s University Hospitals NHS Foundation Hospital, London, UK; 11 Health Economics Research Centre, Nuffield Department of Population Health, University of Oxford, Oxford, UK; 12 Department for Health, University of Bath, Bath, UK

**Keywords:** Stress echocardiography, Cost saving analysis, Health economics, Coronary artery disease, Cardiovascular disease

## Abstract

**Background:**

Stress echocardiography is widely used to detect coronary artery disease, but little evidence on downstream hospital costs in real-world practice is available. We examined how stress echocardiography accuracy and downstream hospital costs vary across NHS hospitals and identified key factors that affect costs to help inform future clinical planning and guidelines.

**Methods:**

Data on 7636 patients recruited from 31 NHS hospitals within the UK between 2014 and 2020 as part of EVAREST/BSE-NSTEP clinical study, were used. Data included all diagnostic tests, procedures, and hospital admissions for 12 months after a stress echocardiogram and were costed using the NHS national unit costs. A decision tree was built to illustrate the clinical pathway and estimate average downstream hospital costs. Multi-level regression analysis was performed to identify variation in accuracy and costs at both patient, procedural, and hospital level. Linear regression and extrapolation were used to estimate annual hospital cost-savings associated with increasing predictive accuracy at hospital and national level.

**Results:**

Stress echocardiography accuracy varied with patient, hospital and operator characteristics. Hypertension, presence of wall motion abnormalities and higher number of hospital cardiology outpatient attendances annually reduced accuracy, adjusted odds ratio of 0.78 (95% CI 0.65 to 0.93), 0.27 (95% CI 0.15 to 0.48), 0.99 (95% CI 0.98 to 0.99) respectively, whereas a prior myocardial infarction, angiotensin receptor blocker medication, and greater operator experience increased accuracy, adjusted odds ratio of 1.77 (95% CI 1.34 to 2.33), 1.64 (95% CI 1.22 to 2.22), and 1.06 (95% CI 1.02 to 1.09) respectively. Average downstream costs were £646 per patient (SD 1796) with significant variation across hospitals. The average downstream costs between the 31 hospitals varied from £384–1730 per patient. False positive and false negative tests were associated with average downstream costs of £1446 (SD £601) and £4192 (SD 3332) respectively, driven by increased non-elective hospital admissions, adjusted odds ratio 2.48 (95% CI 1.08 to 5.66), 21.06 (95% CI 10.41 to 42.59) respectively. We estimated that an increase in accuracy by 1 percentage point could save the NHS in the UK £3.2 million annually.

**Conclusion:**

This study provides real-world evidence of downstream costs associated with stress echocardiography practice in the UK and estimates how improvements in accuracy could impact healthcare expenditure in the NHS. A real-world downstream costing approach could be adopted more widely in evaluation of imaging tests and interventions to reflect actual value for money and support realistic planning.

**Supplementary Information:**

The online version contains supplementary material available at 10.1186/s44156-023-00020-1.

## Introduction

Coronary artery disease (CAD) is a leading cause of morbidity and mortality in the UK and remains a major financial healthcare burden [1]. Early diagnosis is important to prevent acute events and a number of tests and imaging modalities are available, all with relatively similar levels of predictive accuracy [2–5]. In the UK, the National Institute for Health and Care Excellence (NICE) provides guidance on best practice, taking into account economic evidence from cost-effectiveness analysis. Current guidelines recommend non-invasive anatomical imaging as first-line investigation [6]. However, the authors highlighted this guidance was limited by a lack of meaningful data to evaluate real-world downstream costs associated with different imaging tests. Short term, de novo health economic models with instant time horizons, considering only the imaging test and associated complications were used, without ongoing management costs [6]. Furthermore, the imaging combinations modelled did not reflect real world practice and the substantial economic costs of installing new infrastructure to deliver this guidance across the UK were not considered [7, 8].

Stress echocardiography is one of the most widely used functional tests for detecting CAD in the UK [9] and has been shown to be accurate and cost-effective [10–14]. Additionally, the European Society of Cardiology (ESC) guidelines recommend both non-invasive functional imaging and non-invasive anatomical imaging for diagnosis of severe CAD [15]. We have recently reported results from the largest, prospective, observational study of stress echocardiography in the UK (Echocardiography Value and Accuracy at Rest and Stress—EVAREST), which showed stress echocardiography is being performed with a high level of accuracy in the NHS [5, 16]. As all patients in EVAREST are followed up for at least 12 months, we have now evaluated real world downstream costs associated with the CAD patient care pathway. The aim of this costings sub-study is to determine to what extent stress echocardiography accuracy and downstream hospital costs vary across National Health Service (NHS) hospitals in the UK and identify key factors that might be able to be modified to reduce costs within the NHS and help inform future clinical planning and guidelines.

## Methods

### Patient recruitment and follow-up

The EVAREST study (NCT03674255) is an ongoing, prospective, multi-centre, observational study examining the use, accuracy and downstream cost of stress echocardiography in real-world NHS settings, and since 2021, has been conducted in collaboration with the British Society of Echocardiography as the National Review of Stress Echocardiography Practice (BSE-NSTEP). Ethical approval was granted by the Health Research Authority NRES Committee (South Central–Berkshire) review board (IRAS reference:14/SC/1437). Patients are recruited at the time of their stress echocardiogram and are eligible for inclusion if they are aged over 18 years of age and provide written informed consent. The performance and interpretation of the stress echocardiogram is carried out per local centre protocol, and the downstream management of each patient is determined by clinicians at the recruiting centre as per usual care basis. Information relating to patient demographics, stress echocardiogram protocol and stress echocardiogram result are extracted from hospital records. Patients in this analysis were followed up for 12 months using medical records reviews and patient phone calls conducted by hospital staff to determine whether they had undergone any further cardiac imaging investigations and treatments e.g. initiation of medical therapy and/or revascularization, as well as if they had suffered major cardiac events such as myocardial infarction (MI) or cardiac-related death. Full study design is described elsewhere [5].

### Patient and hospital characteristics

All patients recruited from March 2014 to March 2020 across 28 NHS Trusts in England (comprised of 31 hospitals) who had completed a diagnostic stress echo protocol were used in this analysis. Data at the individual level included socio-demographic characteristics (age and gender) and presence of cardiac risk factors at the time of undergoing a stress echocardiogram including smoking status, body-mass index (BMI), hypertension, hypercholesteremia, peripheral vascular disease, diabetes, family history of premature cardiovascular disease, previous CAD, previous MI, and previous revascularisation. Cardiac medications and resting regional wall motion abnormalities (RWMAs) were also included. Data at the hospital level included socio-economic deprivation based on the Office for National Statistics (ONS) Index of Multiple Deprivation (IMD) rank, number of beds in hospital, cardiology attendances per year, and stress echocardiograms performed per year. Bed number was obtained from NHS England [17], and cardiology attendances per year were obtained from NHS Digital [18]. Information related to annual capacity for stress echocardiography were self-reported by each hospital.

### Definition of predictive accuracy

All clinical data were reviewed by an adjudication committee including at least one accredited cardiologist, blinded to stress echocardiogram result and a binary (cardiac/non-cardiac) outcome assigned. Cardiac outcome was defined as angiography demonstrating an anatomically or functionally significant lesion [defined as greater than 70% narrowing (or 50% in the left main stem) or abnormal fractional flow reserve or instantaneous wave-free ratio], referral for revascularization, initiation of appropriate pharmacological therapy, acute coronary syndrome, or cardiac-related death. All patients in whom no additional cardiac intervention, management, or investigation was required were assigned a non-cardiac outcome. A correct stress echocardiogram is categorised as either true positive (TP) or true negative (TN), these are defined as an agreement between the interpretation of the reporting clinician (positive or negative for ischaemia) and the per patient outcome assigned by the study adjudication committee..

### Downstream hospital costs

Cardiac related elective (including day case) and non-elective hospital admissions, as well as further cardiac investigations, of individuals over the 12-month period following their stress echocardiogram were costed using 2019/20 unit costs from the NHS National Schedule of Reference Costs [19]. Where multiple procedure costs were present on the schedule, for example due to multiple complexity and comorbidity scores, a weighted average cost was calculated.

### Statistical analysis

Descriptive statistics (i.e. frequencies, mean, median, standard deviation, and interquartile range) were performed to describe the sample and differences between hospitals were statistically tested using Kruskal–Wallis test for continuous variables and Chi^2^ for categorical variables. Further, a decision tree was constructed using TreeAge Pro Healthcare (TreeAge Software LLC, Massachusetts, USA) to illustrate the management pathway for individuals following a positive or negative stress echocardiogram and calculate the associated mean downstream hospital costs.

Regression analysis was performed to test the association of stress echocardiogram predictive accuracy with non-elective hospitalization and downstream costs. Predictive accuracy was defined in the regression models as an individual having a correct stress echocardiogram or not, a false positive (FP) or not, and a false negative (FN) or not. The latter two determinants of accuracy were used separately in the regression analyses to disentangle their association with downstream hospital costs in the case of a not accurate diagnosis. Total downstream costs per individual over a year were included as an outcome variable alongside a binary variable whether an individual had a non-elective hospital admission for cardiac reasons. The latter outcome variable was specified to test the hypothesis that individuals with FN stress echocardiogram were at higher risk of major adverse cardiovascular events (MACE). We also included variables that were associated with both predictive accuracy and downstream costs (i.e. confounders). These variables included socio-demographic characteristics (age and gender), cardiac risk factors at the time of undergoing a stress echocardiogram (smoking status, BMI, hypertension, hypercholesteremia, peripheral vascular disease, diabetes, family history of premature cardiovascular disease, previous CAD, previous MI, and previous revascularisation), cardiac medications, resting RWMAs, IMD rank, number of beds in hospital, cardiology attendances per year, and stress echocardiograms performed per year. Additional File 1 provides a graphical illustration of the causal pathway with the predictive accuracy as an exposure variable, the predictive accuracy as an outcome variable, and the several confounders at individual and hospital level.

Mixed-effects generalised linear regression models with random intercept and clustered standard errors at hospital level were specified to accommodate the hierarchy of the data (i.e. individuals clustered in hospitals). For binary outcomes, binary distribution with logit function link were used, while for downstream costs gamma distribution and log link were used to accommodate for skewed cost data. Last, regression analysis was used to estimate the annual cost-savings per index stress echocardiogram associated with increasing predictive accuracy. Linear extrapolation was then conducted to estimate the annual cost-savings of increasing predictive accuracy across the EVAREST hospitals, and nationally. For extrapolation at the national level, a reference value of 61,458 stress echocardiograms performed annually at 115 NHS Trusts in the UK as reported by Asher et al. [20], was used. Statistical analysis was carried out using STATA 15-MP (StataCorp, Texas, USA), and used a threshold of 0.05 for statistical significance.

## Results

### Demographics

Follow up data for 12 months following their stress echocardiogram procedure was available for 7636 patients across 28 NHS Trusts (31 hospitals). The median age of the population was 66 (IQR 57 to 73) years and 4278 (56%) were male. There were 1425 (18.7%) individuals with a positive stress echocardiogram, while 6211 (81.3%) had a negative stress echocardiogram. A complete list of patient demographics is shown in Table 1, and aggregated descriptive statistics at the hospital level demonstrate variation in patient characteristics (p < 0.001) [see Additional file 2].

**Table 1:** Patient demographics at the time of stress echocardiogram for all patients (N = 7636). Also shown is the range between the 28 participating NHS Trusts

	Overall cohort (n = 7,636)	Range between 28 NHS Trusts
Median age (years) (IQR)	66 (57–73)	
Mean age (years) (SD)	67.8 (16.6)	55–68 (2.4)
Sex		
Female (%)	3358/7636 (44.0)	11–137 (31.3–57.6)
Male (%)	4278/7636 (56.0)	15–726 (42.4–68.8)
Smoking status		
Non-smoker (%)	3654/7330 (49.9)	13–801 (35.6–65.7)
Ex-smoker (%)	2772/7330 (37.8)	11–308 (18.9–55.2)
Current smoker (%)	904/7330 (12.3)	3–161 (6.1–20.1)
Cardiac risk factors		
Hypertension (%)	3472/7238 (48.0)	13–388 (23.1–76.5)
Hypercholesteremia (%)	2869/7238 (39.6)	6–692 (8.9–76.6)
Peripheral vascular disease (%)	207/7238 (2.9)	0–47 (0–9.4)
Diabetes (%)	1377/7238 (19.0)	5–133 (9.6–35.0)
Family history of premature cardiovascular disease (%)	487/7238 (6.7)	0–98 (0–40.3)
Previous CAD (%)	2773/7568 (36.6)	10–499 (3.4–61.8)
Previous MI (%)	1273/7499 (17.0)	4–245 (2.4–46.8)
Previous CABG (%)	536/7528 (7.1)	0–142 (0–15.5)
Previous stent (%)	1394/7568 (18.4)	2–379 (0.7–32.7)
Medications		
Ace inhibitors (%)	1,298/7,616 (17.0)	4–176 (3.7–41.7)
Angiotensin receptor blocker (%)	580/7,616 (7.6)	0–63 (0–22.4)
Aspirin (%)	2,059/7,616 (27.0)	7–354 (6.5–61.5)
Beta blocker (%)	1,759/7,616 (23.1)	6–279 (5.0–60.4)
Calcium channel blocker (%)	1,181/7,616 (15.5)	3–179 (3.5–39.6)
Nitrates (%)	1,441/7,616 (18.9)	4–134 (2.4–59.4)
Statins (%)	3,462/7,616 (45.5)	17–464 (26.3–78.1)
Resting RWMA (%)	1,092/7,612 (14.4)	1–177 (0.3–34.7)
Deceased (%)	19/7,629 (0.3)	0–5 (0–3.7)

### Predictive accuracy of stress echocardiograpy

Predictive accuracy varied across Trusts with a mean sensitivity and specificity of 81.7% (SD 15.0%, range 40.0–100.0%) and 95.8% (SD 2.7%, range 90.3–100.0%) respectively. Overall accuracy was 94.2% (SD 2.1%, range 89.6–98.2%) [see Additional file 3].

### Variation in downstream costs

**Fig. 1:**
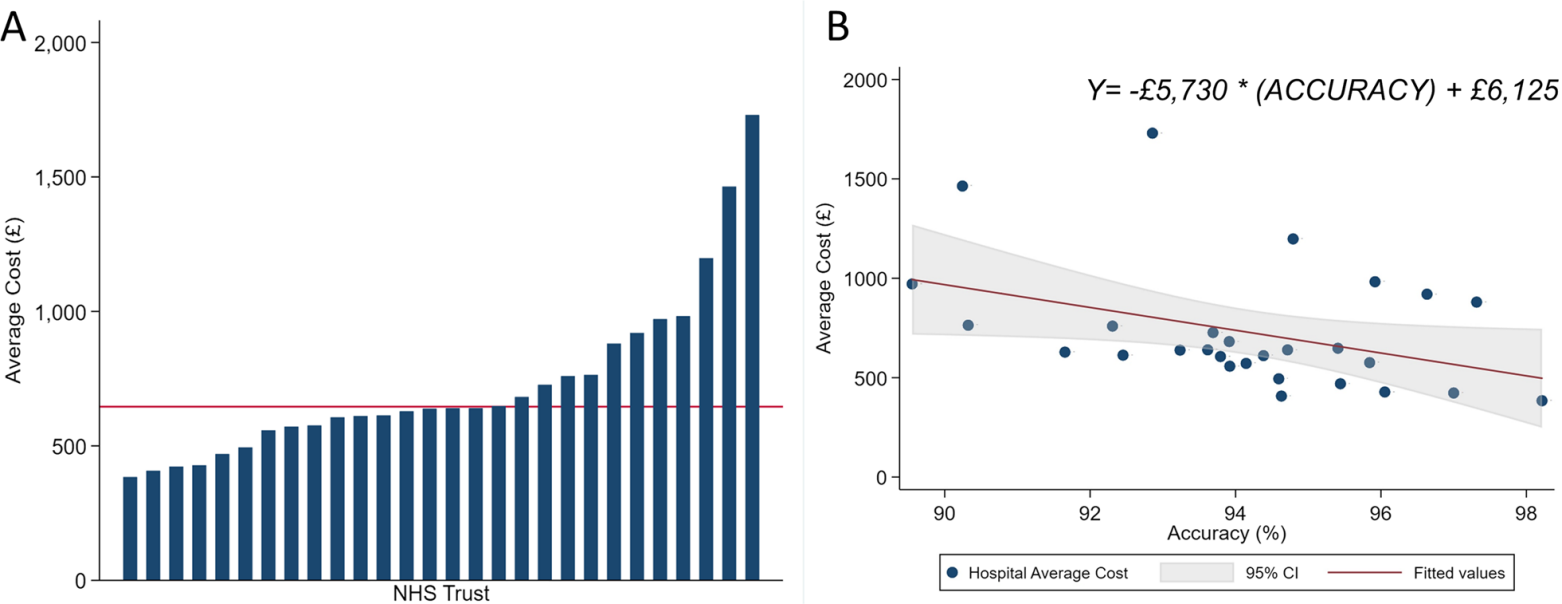
**A** Variation in average downstream cost across NHS Trusts. Red line indicates mean trust cost. **B** Linear regression analysis between predictive accuracy and average downstream costs for each Trust

**Fig. 2:**
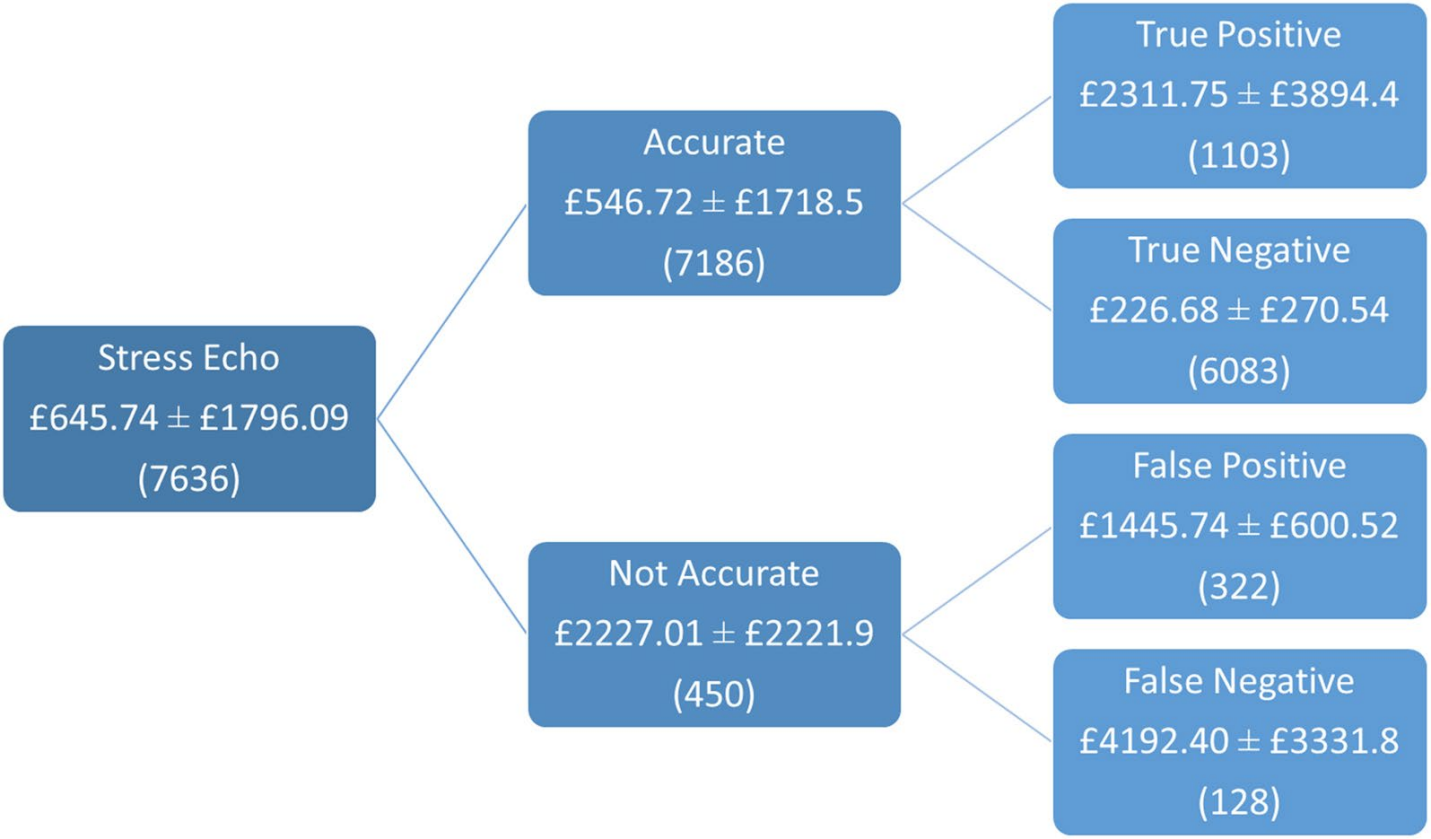
Average downstream costs incurred per patient

The average downstream costs per patient were £646 (SD £1796, median £191, range £191–19,973). The average downstream costs between the 31 hospitals varied from £384–1730 per patient (Fig. 1A). The average hospital cost following a TP stress echocardiogram was £2312 (SD £3894), and £227 (SD £271) after a TN stress echocardiogram. For those with a FP stress echocardiogram, the average hospital cost was £1446 (SD £601), and for a FN stress echocardiogram, the average cost was £4192 (SD £3332) (Fig. 2). A breakdown of outcomes of patient management following a positive and negative stress echocardiogram is given in Table 2. The decision tree depicting all patient downstream outcomes as well as associated costs for a positive and negative stress echocardiogram is shown in Additional file 4 and 5 respectively.

**Table 2:** Breakdown of patient management following positive and negative stress echocardiography

	Positive stress echo	Negative stress echo
No further events/investigations	0	5831
Medical therapy	734	29
Angiographically severe disease	388	124
Revascularisation	295	97
ACS	107	40
Cardiac-related death	3	6

### Demographic associations with accuracy

#### Stress echocardiogram correct

Individuals with hypertension and those with resting RWMAs were associated with a decreased likelihood of a correct stress echocardiogram with an adjusted odds ratio of 0.78 (95% CI 0.65 to 0.93, p < 0.01) and 0.27 (95% CI 0.15 to 0.48, p < 0.001), respectively. Those with a prior MI and those taking an angiotensin receptor blocker were associated with an increased likelihood of a correct stress echocardiogram with an adjusted odds ratio of 1.77 (95% CI 1.34 to 2.33, p < 0.001) and 1.64 (95% CI 1.22 to 2.22, p < 0.01), respectively. In terms of hospital demographics, number of stress echocardiograms performed per year was associated with a slight increased likelihood of a correct stress echocardiogram, adjusted odds ratio 1.06 (95% CI 1.02 to 1.09, p < 0.01). The number of cardiology attendances per year at each hospital, used as a surrogate marker of cardiology department size was associated with a slight decreased likelihood of a correct stress echocardiogram, adjusted odds ratio 0.99 (95% CI 0.98 to 0.99, p < 0.001) [see Additional file 6A].

#### False diagnoses

BMI, hypertension, resting RWMAs, and cardiology attendances were all associated with an increased likelihood of a FP stress echocardiogram, adjusted odds ratio 1.02 (95% CI 1.01 to 1.04, p < 0.01), 1.28 (95% CI 1.02 to 1.60, p < 0.01), 4.68 (95% CI 1.96 to 11.17, p < 0.01), 1.02 (95% CI 1.01 to 1.03, p < 0.01), respectively. Conversely, male sex, diabetes, previous MI, and angiotensin receptor blocker use, were associated with a decreased likelihood of a FP stress echocardiogram, adjusted odds ratio 0.70 (95% CI 0.52 to 0.94, p < 0.05), 0.65 (95% CI 0.44 to 0.97, p < 0.05), 0.44 (95% CI 0.29 to 0.67, p < 0.001), 0.71 (95% CI 0.51 to 0.99, p < 0.05), respectively. Male sex, diabetes, and resting RWMAs, were all associated with an increased likelihood of a FN stress echocardiogram, adjusted odds ratio 1.94 (95% CI 1.32 to 2.86, p < 0.01), 2.02 (95% CI 1.33 to 3.06, p < 0.01), 2.15 (95% CI 1.42 to 3.26, p < 0.001), respectively. No variables were associated with a decrease in the likelihood of a FN stress echocardiogram [see Additional file 6B].

### Hospital admission

#### Non-elective procedures

In the 12-month period following their stress echocardiogram, 162 (2.1%) patients were admitted with an acute coronary syndrome (ACS), 156 (2.0%) received a non-elective invasive coronary angiogram (ICA), 94 (1.2%) were managed medically, 45 (0.6%) had a non-elective percutaneous coronary intervention (PCI), and 4 (0.1%) had a non-elective coronary artery bypass graft (CABG). A further 13 (0.2%) patients underwent both non-elective PCI and CABG. The remaining six patients who did not undergo non-elective coronary angiography were referred directly for non-elective CABG. A correct stress echocardiogram was associated with a decrease in the likelihood of a non-elective hospital admission, adjusted odds ratio 0.13 (95% CI 0.06 to 0.25, p < 0.001) [see Additional file 7]. A false positive or false negative stress echocardiogram was associated with an increased likelihood of non-elective hospital admission, adjusted odds ratio 2.48 (95% CI 1.08 to 5.66, p < 0.05), 21.06 (95% CI 10.41 to 42.59, p < 0.001), respectively [see Additional file 8].

### Associations of accuracy with downstream costs

**Fig. 3:**
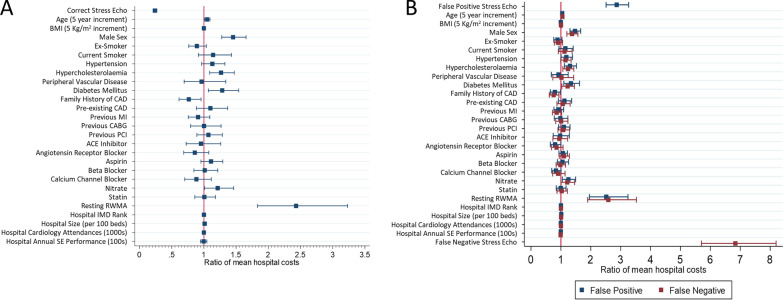
Association of diagnosis with hospital 12-month follow up costs in correct diagnosis (**A**) and false diagnosis (**B**)

A correct stress echocardiogram was associated with 76% less mean downstream cost per patient, (adjusted means ratio: 0.24, 95% CI 0.21 to 0.27; p-value < 0.001) (Fig. 3A) or £1096 (95% CI £912–1280, p < 0.001) compared to an incorrect stress echocardiogram. A FP stress echocardiogram was associated with a 186% increase in mean downstream costs per patient (adjusted means ratio: 2.86, 95% CI 2.51 to 3.27; p-value < 0.001) or £803 (95% CI £646 to 960, p < 0.001), while a FN stress echocardiogram was also associated with a 584% increase in mean downstream costs per patient, (adjusted means ratio: 6.84; 95% CI 5.70 to 8.20; p-value < 0.001) or £1425 (95% CI £1195 to 1654, p < 0.001) (Fig. 3B).

### Cost savings by increasing accuracy

As shown in Fig. 1B, an increase in stress echocardiogram accuracy by 1 percentage point could save on average £57.30 downstream hospital costs in 12 months following a stress echocardiogram. This could be translated to £1 098 211 savings annually across the 31 hospitals in the EVAREST study (19,166 combined stress echocardiograms performed per year self-reported by hospital). A 1 percentage point increase in stress echocardiogram accuracy extrapolated nationally could result in an annual saving of £3.5 million (assuming the EVAREST cohort is representative of UK practice overall). Additionally, 15 hospitals in EVAREST performed below the calculated mean hospital predictive accuracy (94.2%). Increasing stress echocardiogram accuracy at these hospitals to the mean accuracy level in EVAREST would result in an annual cost savings of £772 871. Again, extrapolating to the national level would result in increases in accuracy at 58 hospitals across the UK with a potential cost-saving of £3.2 million.

## Discussion

In this study, we calculated the mean downstream hospital cost over a 12-month period following a stress echocardiogram to be £646 per patient with a variation of £384 and £1730 between the 31 hospitals in our study. This variation in cost is primarily explained by the range in predictive accuracy of stress echocardiogram between centres observed in this real-world setting, which is strongly associated with the increased cost attributed to non-elective hospital admissions. If overall accuracy could be increased by 1 percentage point, then NHS hospitals could save £57.30 per individual undergoing a stress echocardiogram. For NHS budget holders, this would be a cost saving of approximately £3.5 million per year.

### Associations with accuracy

Our findings show people with diabetes and males are more likely to have a FN stress echocardiogram, which mirrors findings from a 2016 study by Premarante et al. [21] and a small cohort study by Elhendy et al. [22]. These findings may be attributed to an increased prevalence of CAD amongst males or a difference in myocardial response to stress. Furthermore, our results show that a higher BMI, female sex, and being non-diabetic are associated with a higher risk of a FP stress echocardiogram, consistent with previous studies examining risk factor associations with FP stress echocardiogram results [23–26]. Those with prior MI and those currently taking angiotensin receptor blockers, perhaps due to a higher pre-test probability of disease in these patients, had a reduced risk of FP stress echocardiogram. It is surprising that angiotensin receptor blockers were the only cardiac medications observed to affect accuracy. A possible explanation for their reduction in false positive rate may be related to their anti-hypertensive effect, thereby reducing hypertension-induced wall motion abnormalities [27]. However, it is not clear why other anti-hypertensive medications did not also affect accuracy and further work is needed to explore this hypothesis. We have previously reported in a smaller sample of the EVAREST dataset that the presence of resting RWMAs was associated with a reduction in specificity and overall accuracy over a six-month follow-up period [5]. This was also evident in the current dataset and the increase in FP stress echocardiogram is likely due to the difficulty in determining whether the resting RWMA has worsened at higher heart rates. An older study by Marcovitz and Armstrong demonstrated an increase in FP rates in the presence of resting RWMAs [28] and our data suggest this continues to be an issue despite newer ultrasound technologies with higher resolutions and frame rates. The finding that prior MI leads to a reduction in FP rate while the presence of RWMAs leads to an increase in FP rate is interesting, since a prior MI is likely to be associated with RWMAs. This discordance suggests the increase in FP rate in those with RWMAs is driven by referral for angiography in those with RWMA but without a history of prior MI. One possible explanation is that the operator has a lower threshold for referral in those with RWMAs without prior cardiac history. This group then is found to either have non-cardiac reasons for their RWMA or their coronary disease is not flow limiting. Surprisingly, a higher number of hospital cardiology attendances was associated with a decreased likelihood of a correct stress echocardiogram. This could reflect similar problems to those observed in Emergency Departments where increased workload leads to poorer patient outcomes [29]. Employing newer technologies including automated reading of stress echocardiograms with artificial intelligence may also prove useful in increasing accuracy. We recently reported [31] a mean increase in sensitivity and specificity of 10% and 1.4% respectively could be achieved when clinicians were provided with an artificial intelligence-based assessment of stress echocardiogram images during a randomised reader study. The PROTEUS randomised controlled trial is currently ongoing to assess the impact of using artificial intelligence-based assessments as a diagnostic aid during stress echocardiography [32]. A real-world costing analysis will be possible in PROTEUS to evaluate whether improvements in accuracy lead to the health economic benefits estimated in this current work.

### Hospital admissions

The significance of FP stress echocardiograms has been debated with some discharging these patients from further investigation following angiography, whilst others advocate for additional scrutiny and management [33, 34]. Our results support the latter argument as we found an increased likelihood of a subsequent non-elective hospital admission following a FP stress echocardiogram. These results are consistent with those of From et al., where all-cause mortality was similar for those patients with TP and FP stress echocardiogram results [25]. This has been further demonstrated recently by Gurunathan et al. [35] who reported similar cardiovascular event rates for patients with a FP and TP stress echocardiogram result even when conducting a subsequent fractional flow reserve investigation. Similarly, Gilchrist et al. [36] reported a significant increase in the likelihood of a major cardiac event for patients with a FP stress echocardiogram when compared to matched controls. Whilst these patients might benefit from increased surveillance, the overall costs associated with a FP stress echocardiogram were still lower than those attributed to FN stress echocardiogram. Whilst the proportion of FN stress echocardiograms accounted for only 1.7% of the total stress echocardiograms performed in this study, the high number of ICAs and rates of PCI in this group resulted in significantly higher costs. Furthermore, a large increase in downstream costs associated with a FN stress echocardiogram related to non-elective admissions for an acute coronary syndrome—one of the most expensive care pathways costed. However, in our multivariate model we did not identify factors that could be addressed to specifically reduce FN stress echocardiogram rates and, therefore, this additional cost. Other studies have demonstrated that increased age, male sex, diabetes, smoking status, previous diagnosis of CAD and resting RWMA are associated with increased mortality and/or new CAD lesion despite a negative stress echocardiogram [23, 37]. This discrepancy may result from a different pattern of referral or patient demographic in the EVAREST cohort study or the longer follow-up period used in these other studies. Long-term follow up of the EVAREST cohort beyond 12-months will be of interest to explore this further.

These results contrast with the assumptions included in the economic modelling conducted to support the recent NICE guidelines on stable chest pain where FN and FP stress echocardiograms were considered of equal importance. Our study provides evidence that FN results are far costlier to the NHS (average annual downstream cost of £4192) compared to those incurred due to a FP stress echocardiogram (average annual downstream cost of £1446). Additionally, the sensitivity and specificity of stress echocardiogram reported in our cohort (81.7% and 95.8%, respectively) are higher than the data included in the economic analysis within the NICE guidelines (75.6% and 80.4%, respectively).

### Strengths of study

This is the first study to provide a detailed examination of care pathway and associated costs over 12 months following stress echocardiogram for a large volume of patients across 28 NHS Trusts (31 hospitals). The data collected in the study consists of a wide variation of patient and organisational characteristics across England, which is likely representative of stress echocardiogram practice across the country. Notably, our data is not modelled and represent true costs down all diagnosis pathways (TN, TP, FN, FP) which has strengths over the instant time-horizon modelling used in the 2016 NICE guideline de novo health economic model. This provides a more holistic view of overall costs as opposed to cost per correct diagnosis reported in the de novo model. Thus, we were able to address assumptions made in the de novo model such as illustrating that there is a marked increase in downstream costs following a FN stress echocardiogram result as compared to costs associated with a FP result. Additionally, our modelling estimated cost-savings associated with national improvement of stress echocardiography accuracy, potentially providing evidence in favour of implementing strategies to improve accuracy at the hospital level and across the UK.

### Limitations to study

In this study, patients were only followed up for a 12-month period. Thus, we may have missed some delayed non-elective hospital admissions. Furthermore, due to the real-world nature of the study, angiography was not performed in all patients to definitively confirm the presence or absence of obstructive CAD. As such, in the case of the absence of angiography, an outcome was assigned based on the stress echocardiogram result and clinical status of the patient during the follow-up period, using methods designed to assign outcomes with missing data [38, 39]. However, the statistical risk of misclassifying a stress echocardiogram as FN when disease was, in fact, present is arguably minimised by the risk of misclassification of stress echocardiograms as FP when disease is not present. Additionally, the costs utilised in this analysis rely on NHS cost codes rather than actual costs to each hospital. Also, due to the nature of the consent process there may be a selection bias amongst the study population compared with other studies using registry or audit data. Finally, while this observational study provides data regarding downstream cost of stress echocardiography, it is unable to provide data on cost effectiveness compared with other clinical management approaches. Given the findings of the ISCHEMIA study, future prospective randomized controlled trials would be of interest to evaluate the role of imaging in decision making and the current manuscript should provide baseline data against which cost savings can be compared.

## Conclusion

Our study provides the first real world downstream costs associated with performance of stress echocardiography. The analysis identified which individuals were at a higher risk of an incorrect stress echocardiogram (notably male sex, hypertension, diabetes, and presence of resting RWMAs) within a broad representative population of England and therefore may require more detailed attention during imaging tests. Furthermore, we have identified that provider workload and experience impact accuracy of stress echocardiogram diagnosis in real world practice. This finding highlights the importance of workforce planning and training in delivery of imaging tests Finally, our findings may be used to assess the actual value for money of innovations that increase cardiac imaging accuracy and support realistic planning of the clinical pathway.

## Supplementary Information

**Additional file 1: Figure S1.** Causal pathway of predictive accuracy with downstream costs.

**Additional file 2: Table S1.** Average patient demographics at each hospital.

**Additional file 3: Table S2.** Organisational Demographic Variation.

**Additional file 4: Figure S2A**. Decision tree depicting patient downstream outcomes 12 months post-stress echocardiogram for (A) Negative Stress Echocardiogram.

**Additional file 5: Figure S2B.** Decision tree depicting patient downstream outcomes 12 months post-stress echocardiogram for (B) Positive Stress Echocardiogram.

**Additional file 6: Figure S3.** (A) Factors associated with a correct stress echo; (B) Factors associated with a false stress echo diagnosis.

**Additional file 7: Figure S4.** Association of a correct stress echocardiogram diagnosis with non-elective hospital admissions.

**Additional file 8: Figure S5.** Association of false stress echocardiogram diagnoses with non-elective hospital admissions.

## Data Availability

The relevant data underlying this article will be shared on reasonable request to the corresponding author.

## Abbreviations

BMI: Body Mass Index
BSE-NSTEP: British Society of Echocardiography: National Review of Stress Echocardiography Practice
CABG: Coronary Artery Bypass Graft
CAD: Coronary artery disease
ESC: European Society of Cardiology
FN: False negative
FP: False positive
ICA: Invasive coronary angiogram
IMD: Index of Multiple Deprivation
MACE: Major Adverse Cardiovascular Events
MI: Myocardial infarction
NHS: National Health Service
NICE: National Institute for Health and Care Excellence
ONS: Office for National Statistics
PCI: Percutaneous Coronary Intervention
RWMAs: Regional wall motion abnormalities
TN: True negative
TP: True positive
