# Supplementary figures and images for: Real world hospital costs following stress echocardiography in the UK: a costing study from the EVAREST/BSE-NSTEP multi-centre study
Source: Echo Res Pract. 2023 May 31;10:8. doi: 10.1186/s44156-023-00020-1 (PMC10230715; doi:10.1186/s44156-023-00020-1)

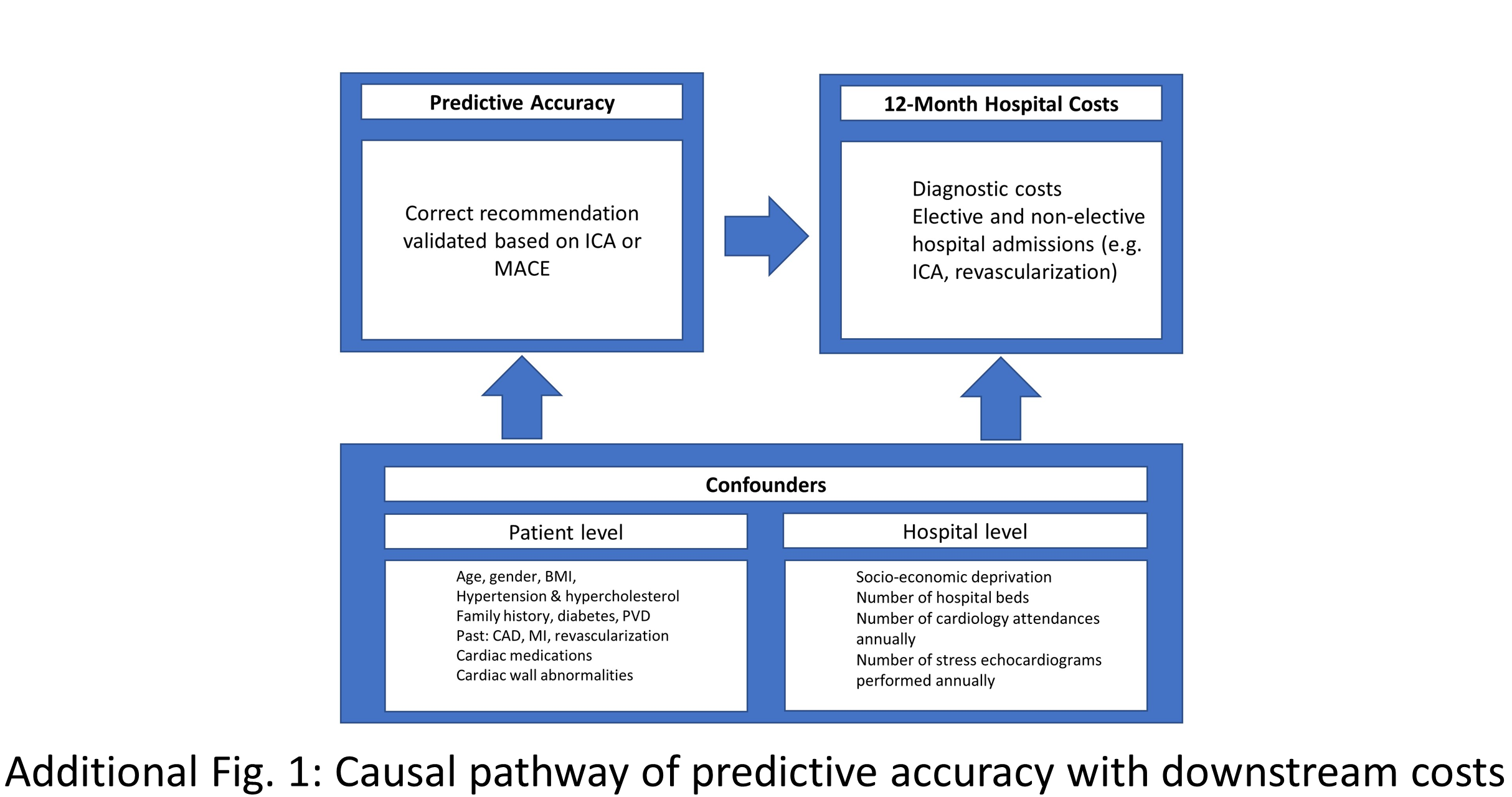

Supplement: Supplementary file 1 — Additional file 1: Figure S1. Causal pathway of predictive accuracy with downstream costs. [file 44156_2023_20_MOESM1_ESM.png]

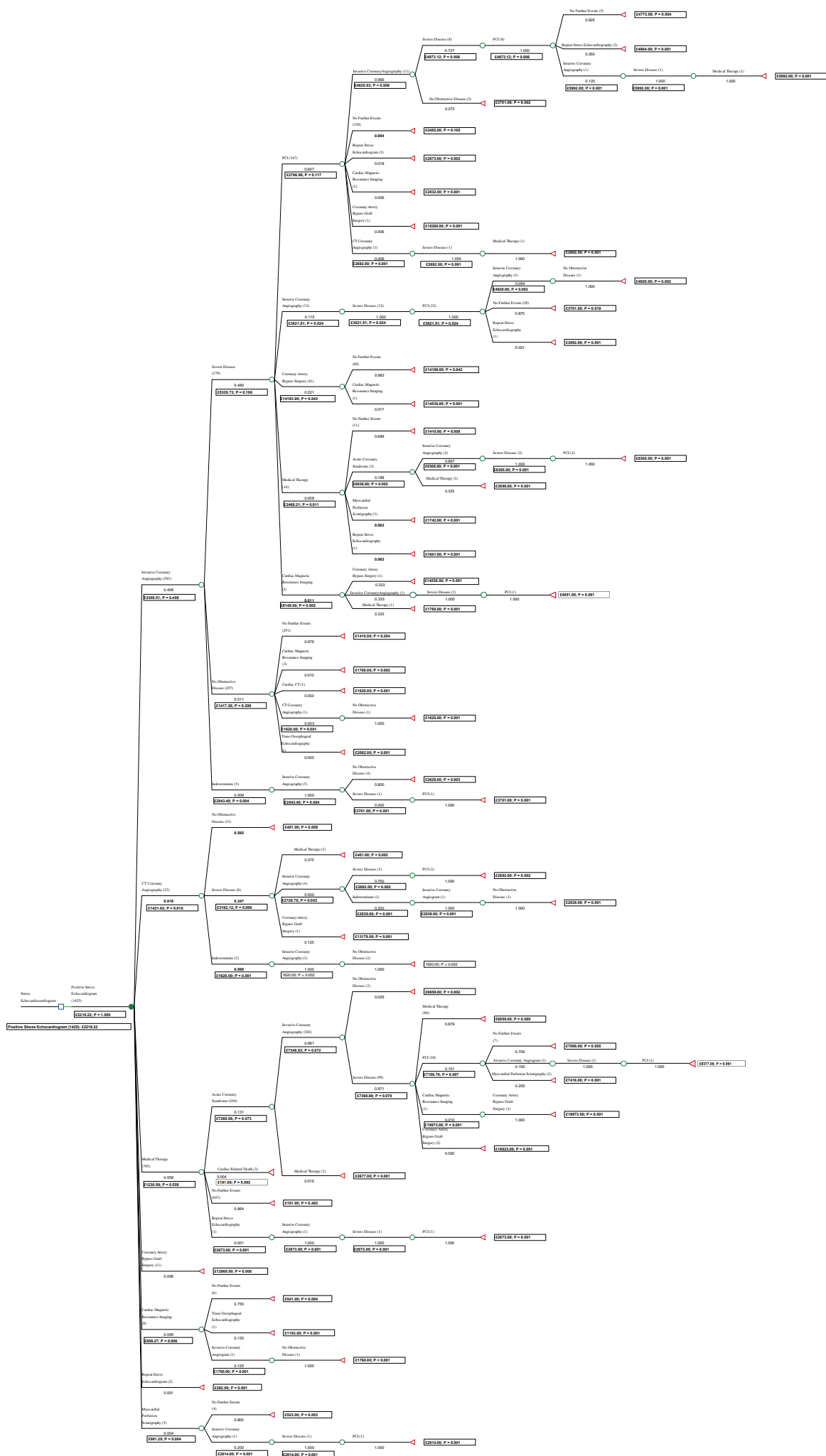

Supplement: Supplementary file 4 — Additional file 4: Figure S2A. Decision tree depicting patient downstream outcomes 12 months post-stress echocardiogram for (A) Negative Stress Echocardiogram. [file 44156_2023_20_MOESM4_ESM.pdf]

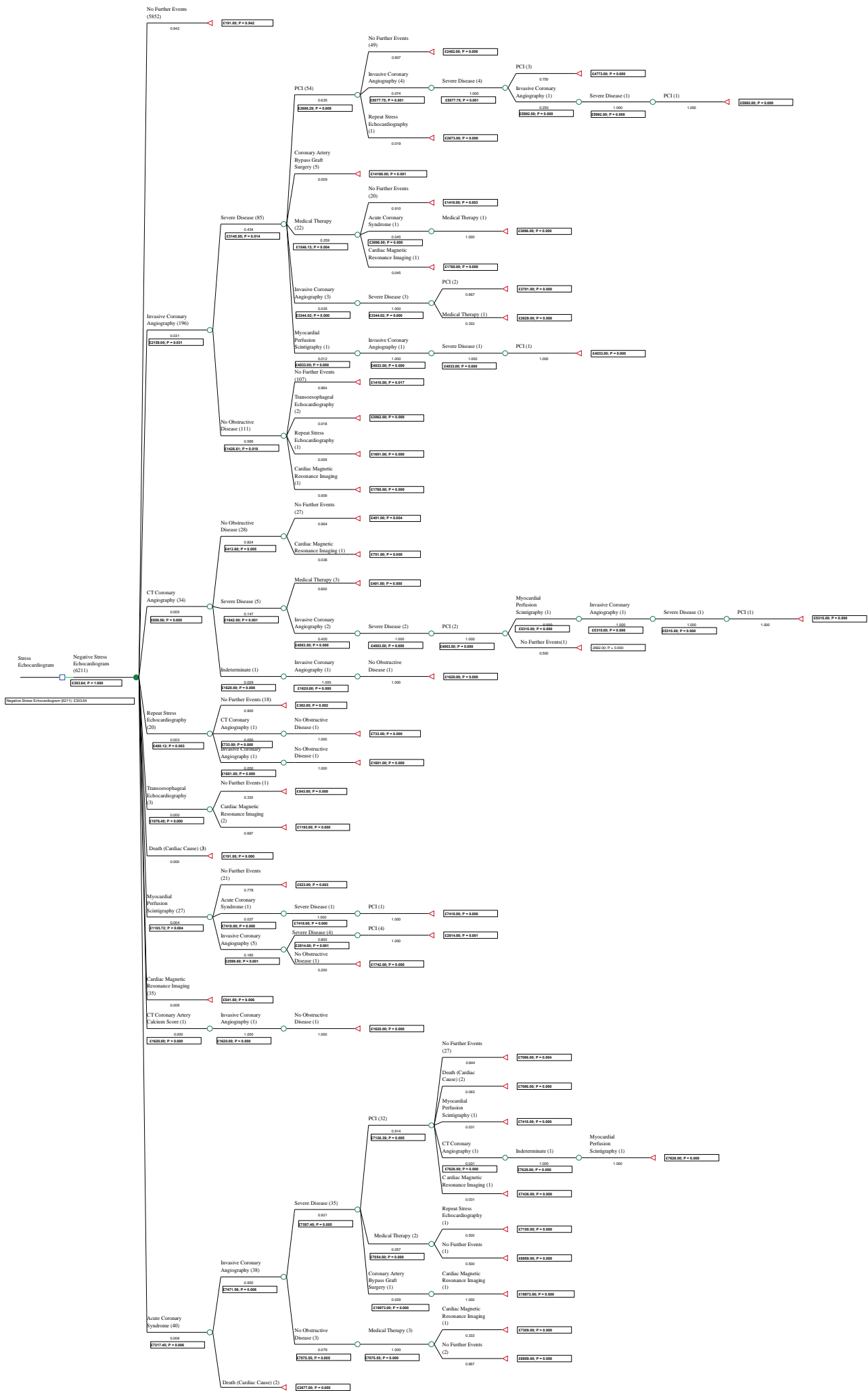

Supplement: Supplementary file 5 — Additional file 5: Figure S2B. Decision tree depicting patient downstream outcomes 12 months post-stress echocardiogram for (B) Positive Stress Echocardiogram. [file 44156_2023_20_MOESM5_ESM.pdf]

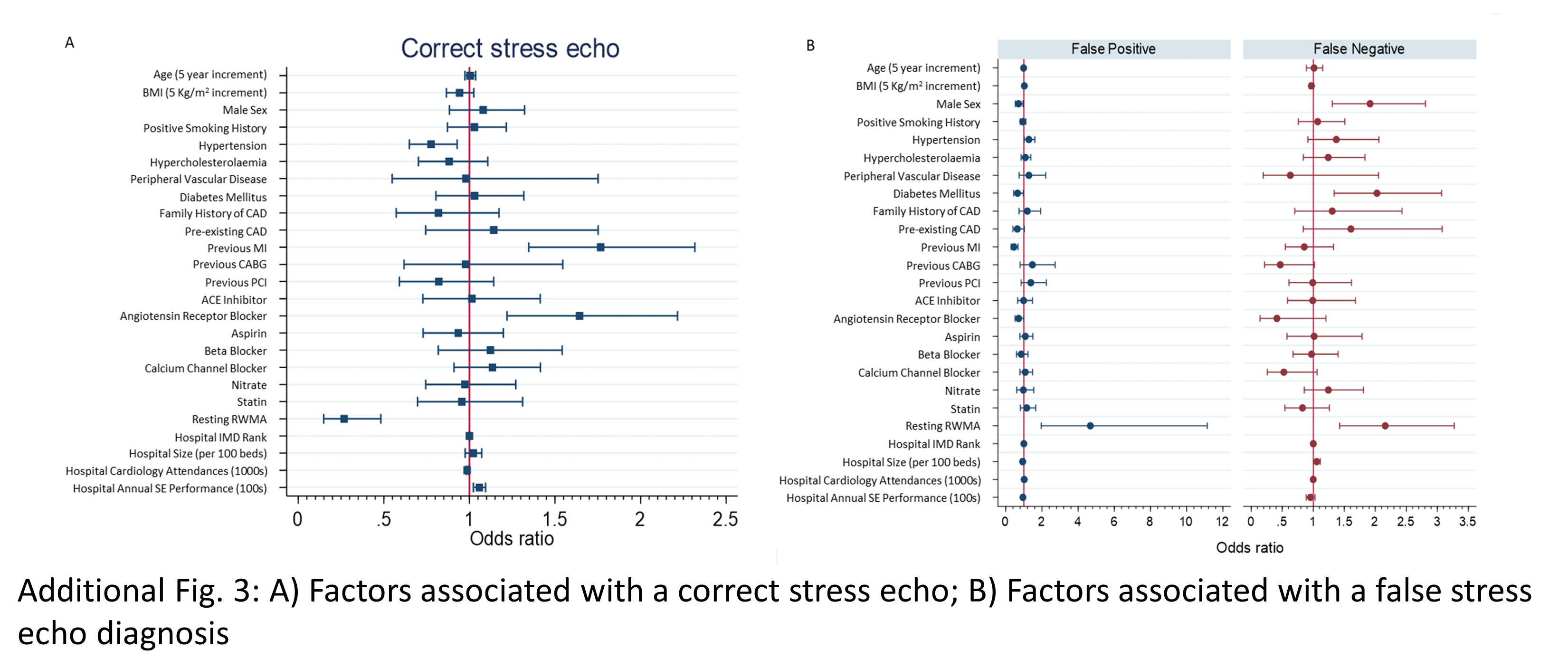

Supplement: Supplementary file 6 — Additional file 6: Figure S3. (A) Factors associated with a correct stress echo; (B) Factors associated with a false stress echo diagnosis. [file 44156_2023_20_MOESM6_ESM.png]

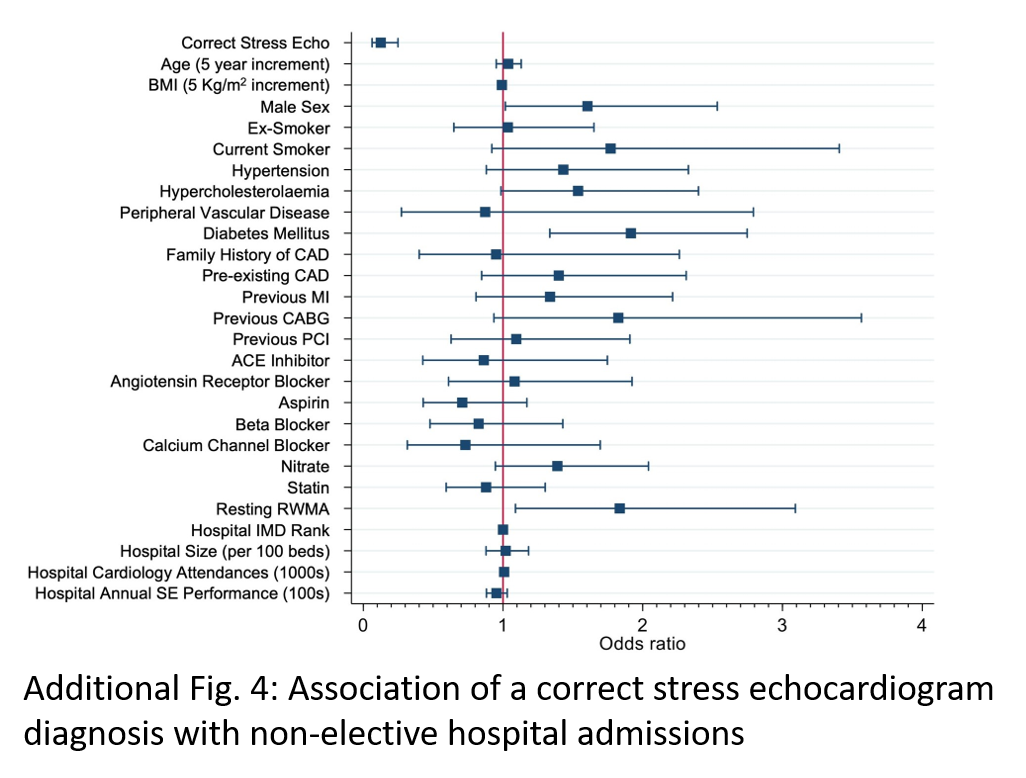

Supplement: Supplementary file 7 — Additional file 7: Figure S4. Association of a correct stress echocardiogram diagnosis with non-elective hospital admissions. [file 44156_2023_20_MOESM7_ESM.png]

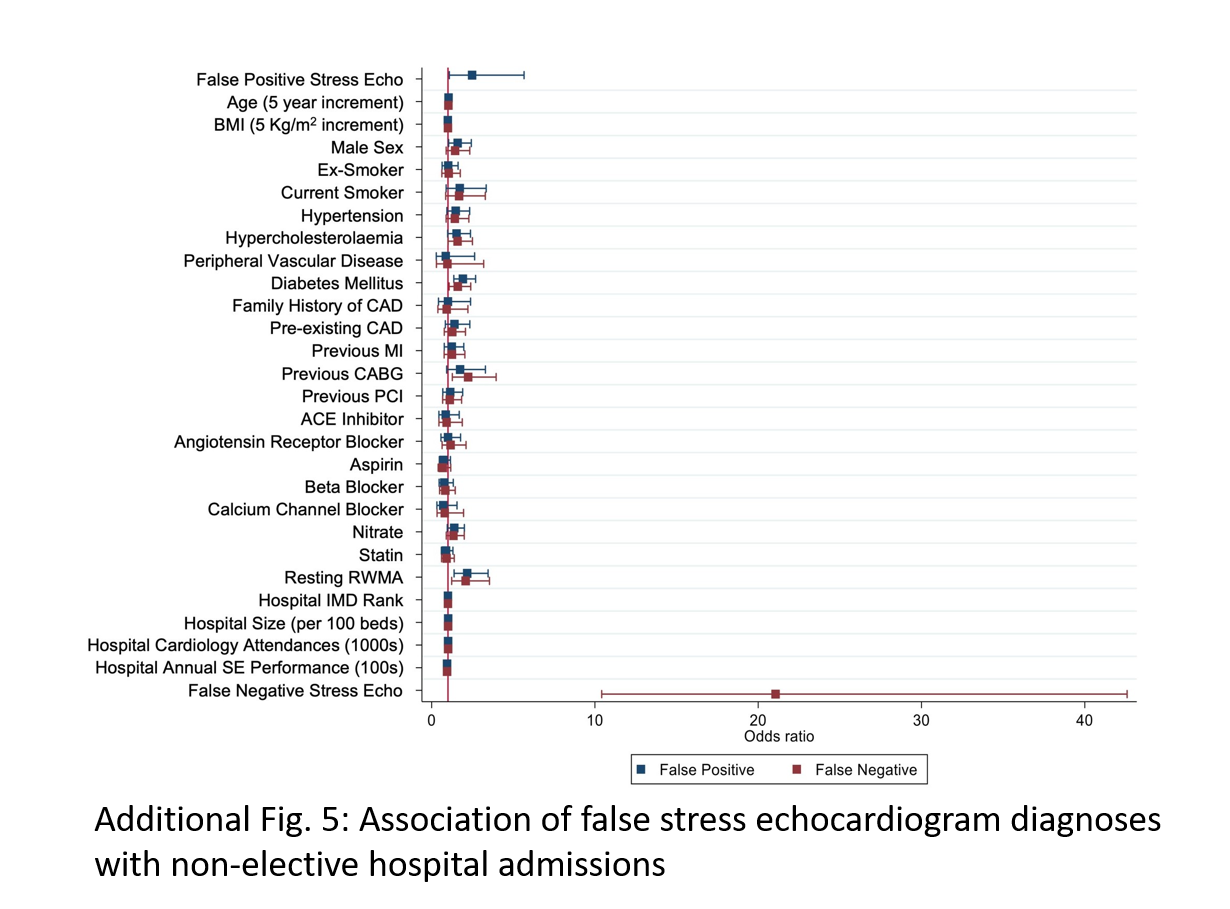

Supplement: Supplementary file 8 — Additional file 8: Figure S5. Association of false stress echocardiogram diagnoses with non-elective hospital admissions. [file 44156_2023_20_MOESM8_ESM.png]
