# Supplementary material for: Real world hospital costs following stress echocardiography in the UK: a costing study from the EVAREST/BSE-NSTEP multi-centre study
Source: Echo Res Pract. 2023 May 31;10:8. doi: 10.1186/s44156-023-00020-1 (PMC10230715; doi:10.1186/s44156-023-00020-1)
Supplement: Supplementary file 2 — Additional file 2: Table S1. Average patient demographics at each hospital. [file 44156_2023_20_MOESM2_ESM.docx]

Supplementary Table 1: Average patient demographics at each hospital

|  | NHS Trusts (N=28) | Range Between NHS Trusts |
| --- | --- | --- |
| Median Age, Years (IQR) | 65.0 (3.3) | 56.0 – 68.0 (13.0 – 20.0) |
| Mean Age, Years (SD) | 64.1 (2.4) | 55.2 – 68.0 (8.9 – 12.9) |
| Median BMI, Kg/m^2^ (IQR) | 28.5 (1.2) | 26.2 – 29.4 (4.3 – 9.8) |
| Mean BMI, Kg/m^2^ (SD) | 28.8 (0.9) | 26.5 – 30.1 (3.7 – 7.6) |
| *Sex* |  |  |
| Female, N (%) | 120 (43.3) | 11 – 544 (31.3 – 57.6) |
| Male, N (%) | 153 (56.7) | 15 – 726 (42.4 – 68.8) |
| *Smoking Status* |  |  |
| Non-Smoker, N (%) | 131 (48.2) | 13 – 801 (35.6 – 65.7) |
| Ex-Smoker, N (%) | 99 (40.0) | 11 – 308 (18.9 – 55.2) |
| Current Smoker, N (%) | 32 (11.8) | 3 – 161 (6.1 – 20.1) |
| *Cardiac Risk Factors* |  |  |
| Hypertension, N (%) | 124 (52.4) | 13 – 388 (23.1 – 76.5) |
| Hypercholesteremia, N (%) | 102 (37.6) | 6 – 692 (8.9 – 76.6) |
| Peripheral Vascular Disease, N (%) | 7 (2.8) | 0 – 47 (0.0 – 9.4) |
| Diabetes, N (%) | 40 (20.9) | 5 – 133 (9.6 – 35.0) |
| Family history of premature cardiovascular disease, N (%) | 17 (7.9) | 0 – 98 (0.0 – 40.3) |
| Previous CAD, N (%) | 99 (37.5) | 10 – 499 (3.4 – 61.8) |
| Previous MI, N (%) | 45 (17.8) | 4 – 245 (23.4 – 46.8) |
| Previous CABG, N (%) | 19 (6.0) | 0 – 142 (0.0 – 15.5) |
| Previous Stent, N (%) | 50 (17.6) | 2 – 379 (0.7 – 32.7) |
| *Medications* |  |  |
| ACE Inhibitors, N (%) | 46 (18.9) | 4 – 176 (3.7 – 41.7) |
| Angiotensin Receptor Blocker, N (%) | 21 (8.8) | 0 – 63 (0.0 – 22.4) |
| Aspirin, N (%) | 74 (29.1) | 7 – 354 (6.5 – 61.5) |
| Beta Blocker, N (%) | 63 (26.4) | 6 – 279 (5.0 – 60.4) |
| Calcium Channel Blocker, N (%) | 42 (16.8) | 3 – 179 (3.5 – 39.6) |
| Nitrates, N (%) | 51 (22.2) | 4 – 134 (2.4 – 59.4) |
| Statins, N (%) | 124 (48.2) | 17 – 464 (26.3 – 78.1) |
| Resting RWMA, N (%) | 39 (15.0) | 1 – 177 (0.3 – 34.7) |
| Deceased, N (%) | 1 (0.4) | 0 – 5 (0.0 – 3.7) |
