# Supplementary material for: Real world hospital costs following stress echocardiography in the UK: a costing study from the EVAREST/BSE-NSTEP multi-centre study
Source: Echo Res Pract. 2023 May 31;10:8. doi: 10.1186/s44156-023-00020-1 (PMC10230715; doi:10.1186/s44156-023-00020-1)
Supplement: Supplementary file 3 — Additional file 3: Table S2. Organisational Demographic Variation. [file 44156_2023_20_MOESM3_ESM.docx]

Supplementary Table 2 Organisational Demographic Variation

| Variable | N | Mean | SD | P50 | IQR | Min | Max |
| --- | --- | --- | --- | --- | --- | --- | --- |
| Sensitivity | 28 | 0.816561 | 0.150108 | 0.871795 | 0.20311 | 0.4 | 1 |
| Variable | N | Mean | SD | P50 | IQR | Min | Max |
| Specificity | 28 | 0.958214 | 0.027462 | 0.95786 | 0.039659 | 0.903846 | 1 |
| Variable | N | Mean | SD | P50 | IQR | Min | Max |
| Accuracy | 28 | 0.941661 | 0.02123 | 0.942646 | 0.025963 | 0.895522 | 0.982143 |
